# Metagenomic Detection of Bacterial Zoonotic Pathogens among Febrile Patients, Tanzania, 2007–2009^1^

**DOI:** 10.3201/eid3008.240529

**Published:** 2024-08

**Authors:** Robert J. Rolfe, Sarah W. Sheldon, Luke C. Kingry, Jeannine M. Petersen, Venance P. Maro, Grace D. Kinabo, Wilbrod Saganda, Michael J. Maze, Jo E.B. Halliday, William L. Nicholson, Renee L. Galloway, Matthew P. Rubach, John A. Crump

**Affiliations:** Duke University Department of Medicine Division of Infectious Diseases and International Health, Durham, North Carolina, USA (R.J. Rolfe, M.P. Rubach, J.A. Crump);; Centers for Disease Control and Prevention, Fort Collins, Colorado, USA (S.W. Sheldon, L.C. Kingry, J.M. Petersen);; Kilimanjaro Christian Medical Centre, Moshi, Tanzania (V.P. Maro, G.D. Kinabo, M.P. Rubach, J.A. Crump);; Kilimanjaro Christian Medical University College, Moshi (V.P. Maro, G.D. Kinabo, J.A. Crump);; Mawenzi Regional Referral Hospital, Moshi (W. Saganda);; University of Otago Department of Medicine, Christchurch, New Zealand (M.J. Maze);; University of Glasgow School of Biodiversity, One Health and Veterinary Medicine, Glasgow, Scotland, UK (J.E.B. Halliday);; Centers for Disease Control and Prevention, Atlanta, Georgia, USA (W.L. Nicholson, R.L. Galloway);; Duke-National University of Singapore Programme in Emerging Infectious Diseases, Singapore (M.P. Rubach);; Duke University Global Health Institute, Durham (M.P. Rubach, J.A. Crump);; Centre for International Health, University of Otago, Dunedin, New Zealand (J.A. Crump)

**Keywords:** Bacteria, vector-borne diseases, zoonoses, bacterial zoonoses, Ehrlichia, Coxiella, Leptospira, Bartonella, metagenomics, Rickettsia, Tanzania, East Africa

## Abstract

Bacterial zoonoses are established causes of severe febrile illness in East Africa. Within a fever etiology study, we applied a high-throughput 16S rRNA metagenomic assay validated for detecting bacterial zoonotic pathogens. We enrolled febrile patients admitted to 2 referral hospitals in Moshi, Tanzania, during September 2007–April 2009. Among 788 participants, median age was 20 (interquartile range 2–38) years. We performed PCR amplification of V1–V2 variable region 16S rRNA on cell pellet DNA, then metagenomic deep-sequencing and pathogenic taxonomic identification. We detected bacterial zoonotic pathogens in 10 (1.3%) samples: 3 with *Rickettsia typhi*, 1 *R. conorii*, 2 *Bartonella quintana*, 2 pathogenic *Leptospira* spp., and 1 *Coxiella burnetii*. One other sample had reads matching a *Neoerhlichia* spp. previously identified in a patient from South Africa. Our findings indicate that targeted 16S metagenomics can identify bacterial zoonotic pathogens causing severe febrile illness in humans, including potential novel agents.

Bacterial zoonoses cause severe febrile illness in East Africa (*1*). Patients with bacterial zoonotic diseases can have nonspecific febrile illnesses that are difficult to diagnose clinically or in the laboratory because many of the pathogens are fastidious or nonculturable. Previous studies have used serologic or molecular methods to confirm leptospirosis, Q fever, spotted fever group rickettsioses (SFGR), and typhus group rickettsioses (TGR) among febrile participants at hospitals in East Africa (*2*–*5*). We previously evaluated participants from 2 cohort studies of febrile inpatients in northern Tanzania by using paired microscopic agglutination test (MAT) and PCR for leptospirosis, and by using paired immunofluorescence antibody (IFA) test for SFGR, TGR, and Q fever (*6*–*9*). In those cohort studies from 2007­–2008 and 2012–2014, the estimated prevalence of acute leptospirosis was 8.8% (2007–2008) and 1.8% (2012–2014), acute SFGR prevalence 8.8% (2007–2008) and 8.9% (2012–2014), and acute Q fever prevalence 4.7% (2007–2008) and 8.1% (2012–2014) (*7*,*8*). Acute TGR was evaluated in 1 of the cohorts, and 2 confirmed infections were identified among 450 febrile participants (*6*).

PCR on acute samples and serologic testing of acute and convalescent samples are currently the most common methods to confirm diagnosis of several bacterial zoonoses (*10*). However, PCR and serology both target known pathogens and neither can identify potential novel pathogens. To characterize novel tickborne pathogens, the Bacterial Diseases Branch, Division of Vector-Borne Diseases, National Center for Emerging and Zoonotic Infectious Diseases, Centers for Disease Control and Prevention (CDC; Fort Collins, CO, USA), developed a 16S rRNA metagenomics assay to evaluate samples for known and novel tickborne and other zoonotic pathogens (*11*). The assay accurately differentiated and identified *Anaplasma*, *Bartonella*, *Borrelia*, *Coxiella*, *Ehrlichia*, *Leptospira*, and *Rickettsia* bacteria (*11*). CDC tested spiked healthy human blood and water specimens, and the assay readily identified zoonotic pathogens among commensal and background organisms commonly amplified when targeting 16S (*11*). In spiked blood specimens, the assay demonstrated equivalent analytic sensitivity to reverse transcription PCR (RT-PCR) but showed some loss of sensitivity when specimens were spiked with multiple pathogenic species (*11*).

In this study, we leveraged that high-throughput 16S rRNA metagenomic assay to interrogate venous blood cell pellets for a broad range of bacterial zoonotic pathogens in a febrile patient study cohort. We assessed whether 16S interrogation could provide insights into causes of febrile illness by detecting novel or under-appreciated pathogens or by genotypic characterization of pathogens already known to cause disease in the study area, such as SFGR and TGR.

## Methods

### Study Procedures and Participants

We performed a prospective cohort study that enrolled pediatric and adult medical patients admitted with fever to 2 referral hospitals in Moshi, Tanzania, duing September 2007–April 2009 (*4*–*6*,*12*,*13*). In brief, infants and children >2 months and <13 years of age admitted to the pediatric ward were eligible if they had a history of fever in the previous 48 hours, an axillary temperature >37.5°C, or a rectal temperature of >38.0°C. Adolescents and adults >13 years of age who were admitted to the adult medicine ward were eligible to participate if they had an oral temperature >38.0°C.

A clinical officer performed a standardized clinical history and physical examination and recorded vital signs. Collected demographic and clinical information included sex, age, and rural or urban residence. 

We drew venous blood samples in EDTA tubes within 24 hours of hospital admission and fractionated samples by centrifugation into plasma and cell pellets. We fractionated blood collected into red top plain tubes into serum, first by gravity on the bench top, then by centrifugation. We performed complete blood counts by using Cell-Dyn 3500 automated hematology analyzer (Abbott Laboratories, https://www.abbott.com). We asked participants to return 4–6 weeks after enrollment to provide a convalescent serum sample. We stored the resulting serum, plasma, and cell pellets at −70°C. We shipped cell pellets on dry ice to CDC for 16S metagenomic analysis. The pellets remained frozen at −70°C after collection and did not go through freeze–thaw cycles until testing. CDC performed testing in 3 different sequencing runs during August 5–December 20, 2021.

We used serology and PCR testing to determine patients’ HIV status. We performed serologic testing by using 2 rapid antibody tests on whole blood, Capillus HIV-1/HIV-2 (Trinity Biotech PLC, https://www.trinitybiotech.com) and Determine HIV-1/2 Ag/AB Combo (Abbott Laboratories). After March 4, 2008, we replaced the Capillus test with the SD Bioline HIV 1/2 Test version 3.0 (Abbott Laboratories). When rapid test results were discordant, we tested the sample by using Vironostika Uni-Form HIV II plus O Ab ELISA (bioMérieux, https://www.biomerieux.com). If the ELISA was positive, we used Genetic Systems HIV-1 Western blot kit (Bio-Rad Laboratories, https://www.bio-rad.com) as a confirmatory test. We obtained HIV-1 RNA by using the Abbott m2000 System RealTime HIV-1 assay (Abbott Laboratories) to diagnose acute HIV infection in seronegative adults and for early HIV diagnosis in infants (*6*,*12*).

As described previously (*4*,*6*–*9*), we shipped serum and plasma on dry ice to CDC’s Bacterial Special Pathogens Branch, Division of High-Consequence Pathogens and Pathology, for MAT for pathogens that cause leptospirosis. We shipped samples to CDC’s Rickettsial Zoonoses Branch, Division of Vector-Borne Diseases, for IFA serologic analyses for pathogens that cause Q fever, SFGR, and TGR. 

CDC performed *Leptospira* real-time PCR on samples from participants with serologically confirmed or probable leptospirosis and on participants who died before providing a convalescent serum sample (*9*). In that population of febrile participants, cases of confirmed acute leptospirosis were defined by a >4-fold increase in MAT titer or detection by real-time PCR; cases of probable leptospirosis had a reciprocal MAT titer >800 and evidence of exposure to pathogenic leptospires as a reciprocal MAT titer >100 (*5*). For seropositive cases, we defined the predominant reactive serogroup as the serovar with the highest MAT titer.

We defined cases of confirmed acute Q fever as active fever and a >4-fold increase in reciprocal IFA titer to the *C. burnetii* phase II antigen. We defined serologic evidence of Q fever exposure as a case with an IFA titer >1,000 to phase I antigen; for patients who did not meet the case definition of a serologic diagnosis, we considered >64 to phase II antigen in either acute or convalescent sample as confirmed acute Q fever (*4*). 

We defined confirmed acute SFGR and TGR cases as presence of fever and >4-fold rise in IFA titer to *R. conorii* for SFGR or *R. typhi* for TGR. For patients who did not meet the case definition for confirmed acute SFGR or TGR, we defined an IFA titer >64 for *R. conorii* as exposure for SFGR and titer of >64 for *R. typhi* as exposure for TGR (*4*).

### 16S rRNA Metagenomic Assay

We performed metagenomic 16S rRNA testing by using previously outlined methods (*1*). In brief, we extracted DNA from cell pellets by using the MagNA Pure 96 instrument (Roche, https://www.roche.com) and the DNA and Viral NA Small-Volume kit with the associated DNA Blood SV 3.1 extraction protocol (Roche) using input and elution volumes of 100 µL. To perform multiplex sequencing on the 788 samples, we amplified the V1–V2 region of the 16S rRNA and added dual Nextera XT indices by using the XT Index Kit v2, sets A–D (Illumina, https://www.illumina.com) to the V1–V2 amplicons. We quantified, normalized to a final concentration of 4 nM, and pooled resulting libraries to enable sequencing of 384 samples in each Illumina MiSeq run. We also included DNA extraction controls, PCR controls, and internal sequencing controls in each run (*8*). Pooled libraries had a final contration of 12.5 pM with 12.5 pM PhiX (10%), which we then sequenced by using MiSeq v2 (500 cycle) reagent kit and MiSeq sequencer (Illumina).

### Bioinformatic Analysis, Taxonomic Prediction, and Phylogenetic Analysis

We performed bioinformatic data processing as previously described (*11*). We demultiplexed sequence reads into individual samples, then used internal MiSeq software to remove the adaptor and indices. We used Kraken 0.10.5 (*14*) and the MiniKraken database (https://ccb.jhu.edu/software/kraken) to assign taxonomic predictions to quality-trimmed, merged reads. We mapped reads from samples with a MiniKraken taxonomic prediction of *Anaplasma*, *Bartonella*, *Coxiella*, *Ehrlichia*, *Leptospira*, or *Rickettsia* to reference sequences by using CLC Genomics Workbench (QIAGEN, https://www.qiagen.com), and tested by BLASTn (https://blast.ncbi.nlm.nih.gov). We did not perform further analyses for reads with taxonomic predictions to other genera, including commensal and background organisms identified in control specimens (i.e., Enterobacterales). We constructed phylogenetic trees in MEGA version 10.0.5 (*15*) by using the maximum-likelihood method and Kimura 2-parameter model with 1,000 bootstrap replicates.

### Statistical Analyses

We performed descriptive data analysis in proportions. We described continuous variables as median and interquartile range (IQR). We performed statistical analyses in R version 4.0.1 (The R Foundation for Statistical Computing, https://www.r-project.org) using the tableone and tidyverse packages (*16*,*17*). We predicated sample size on participant accrual during the parent study enrollment period (*12*,*13*).

### Ethics Considerations

This study was approved by the Kilimanjaro Christian Medical University College Heath Research Ethics Committee (clearance certificates 133 and 138), the Tanzania National Institute for Medical Research Ethics Coordinating Committee (clearance certificates NIMR/HQ/R.8a/Vol.IX/439 and NIMR/HQ/R.8a/Vol.IX/473), and the Institutional Review Board at Duke University Medical Center (protocol nos. 8397 and 8400). CDC acknowledged the study protocol through deferral to the Duke University Medical Center Institutional Review Board. 

Written informed consent was obtained from all participants. A parent or legal guardian provided consent for participants <18 years of age. In addition to consent for study participation, which included permission for future not yet determined analyses on stored blood for HIV research, written informed consent was also obtained from participants for a data and sample repository for future, unspecified research.

## Results

Among 788 febrile illness participants who had a cell pellet available for testing and available sociodemographic data, the median age was 20 (IQR 2–38) years, 239/744 (32.1%) were HIV-infected, 384/766 (50.1%) were male and 382/766 (49.9%) female, and 314/664 (47.3%) lived in an urban setting. Ten (1.3%) participants had 100% sequence identity match in BLASTn for a bacterial zoonotic pathogen detected in cell pellets via 16S metagenomic sequencing: 3 *R. typhi* matched accession no. NC_017066.1, one *R. conorii* matched accession no. NC_003103.1, two *Bartonella quintana* matched accession no. AP019773.1, one *Leptospira borgpetersenii* matched accession no. NZ_CP026671.1, one *L. kirschneri* matched accession no. CP092660.1, and one *C. burnetii* matched accession no. CP014563.1; one sample had reads matching an uncultured and unnamed *Neoehrlichia* sp. previously identified in a patient from South Africa (*18*). Of the 10 participants with a detected bacterial zoonotic pathogen, 5 (50.0%) were male and 5 (50.0%) female; median age was 41 (IQR 32–56) years. One of the 2 participants with detected *B. quintana* was HIV-infected (Table 1). Symptom onset varied from 3–40 days before enrollment, and 7 (70%) patients had symptoms for <7 days. For the 10 samples with detected bacterial zoonotic pathogens, we used BLASTn for taxonomic prediction, read counts, and abundance relative to all other bacterial taxa detected by 16S in clinical samples (Table 2).

**Table 1:** Organisms and patients characteristics in a study of metagenomic detection of bacterial zoonotic pathogens among febrile patients, Tanzania, 2007–2009*

Organism detected	Patient age, y/sex	Rural res.	Illness onset, d	Symptoms					Serologic testing			Results
					Cell count, × 10^3^/µL				*Leptospira *MAT	Q fever IFA	*Rickettsia *IFA	
					WBC†	Plat.	ALC					
*Bartonella quintana*	10/M	Y	4	Fever, dyspnea, convulsions	11.9	89	2.59		Acute and convalescent	ELISA screen negative	Acute and convalescent	Probable acute leptospirosis‡
*B. quintana*	47/M§	N	40	Fever, cough, hemoptysis, dyspnea, weight loss	18.4	124	1.55		ND	ND	ND	NA
*Coxiella burnetii*	22/M	Y	2	Fever, rigors	4.6	93	1.92		Acute only	ND	Acute only	Probable acute leptospirosis‡
*Leptospira borgpetersenii*	43/F	Y	4	Fever, rigors, emesis, headache	4.2	85	0.33		Acute and convalescent	ELISA screen negative	Acute and convalescent	Confirmed acute leptospirosis‡
*L. kirschneri*	60/F	N	3	Fever, emesis, headache	5.4	114	0.52		Acute only	ND	Acute only	NA
*Rickettsia conorii*	70/M	Y	7	Fever, rigors, dyspnea, diarrhea, headache	15.1	118	1.77		ND	ND	ND	NA
*Rickettsia typhi*	36/M	Y	14	Fever, rigors, headache, nuchal rigidity, dysuria	6.6	52	0.92		Acute only	ND	Acute only	Exposure to SFG *Rickettsia* and typhus group *Rickettsia*
*R. typhi*	77/F	N	3	Fever, rigors	4.5	97	0.35		Acute and convalescent	ELISA screen negative	Acute and convalescent	Confirmed acute SFGR
*R. typhi*	31/F	Y	30	Fever, rigors, emesis, headache	7.2	148	0.66		Acute and convalescent	ELISA screen negative	Acute and convalescent	Confirmed acute SFGR; exposure to typhus group *Rickettsia*
*Neoehrlichia *spp.	40/F	N	3	Fever, rigors, hemoptysis, diarrhea, emesis, headache	3.0	25	0.65		ND	ND	ND	NA

*Most patients were HIV-negative; 1 patient with *B. quintana *was HIV-infected. ALC, absolute lymphocyte count; IFA, immunofluorescence assay; MAT, microscopic agglutination testing; NA, not applicable; ND, not done; Plat., platelets; res., resident; SFG, spotted fever group; SFGR, spotted fever group rickettsioses; WBC, white blood cells (leukocytes). †Based on MAT results. ‡*Leptospira* PCR was not performed for this sample. §HIV-positive patient.

**Table 2:** Metagenomic sequencing results used for detection of bacterial zoonotic pathogens among febrile patients, Tanzania, 2007–2009*

Organism	V1–V2 sufficient for species ID	MiniKraken taxonomic prediction†	BLASTn taxonomic prediction (% identity)‡	Sequence in database	Read count	% Abundance
*Bartonella quintana*	Y	*B. quintana*	*B. quintana* AP019773.1 (100)	Y	4,730	98.21
*B. quintana*	Y	*B. quintana*	*B. quintana* AP019773.1 (100)	Y	3,568	38.60
*Coxiella burnetii*	Y	*C. burnetii*	*C. burnetii* CP014563.1 (100)	Y	1,795	47.97
*Leptospira borgpetersenii*	Y	*L. borgpetersenii*	*L. borgpetersenii* CP047520.1 (100)	Y	18,903	94.41
*L. kirschneri*	Y	*Leptospira* spp.	*L. kirschneri* CP092660.1 (100)	N	28,008	99.32
*Rickettsia conorii*	Y	*Rickettsia* spp.	*R. conorii* MG564258.1 (100)	N	159	28.29
*R. typhi*	Y	*Rickettsia typhi*	*R. typhi* LS992663.1 (100)	Y	429	47.72
*R. typhi*	Y	*Rickettsia typhi*	*R. typhi* LS992663.1 (100)	Y	7,274	82.02
*R. typhi*	Y	*Rickettsia typhi*	*R. typhi* LS992663.1 (100)	Y	9,527	83.16
*Candidatus* Neoehrlichia spp.	Y	Anaplasmataceae, *Ehrlichia ruminantium*, *Ehrlichia*	Uncultured *Candidatus* Neoehrlichia sp. SA1 OP208838.1 (100)	N	40,238	98.56

*ID, identification; V1–V2, variable regions 1 and 2. †Johns Hopkins University Center for Computational Biology (https://ccb.jhu.edu/software/kraken). ‡National Center for Biotechnology Information taxonomy and accession nos. from BLASTn (https://blast.ncbi.nlm.nih.gov).

**Figure 1:**
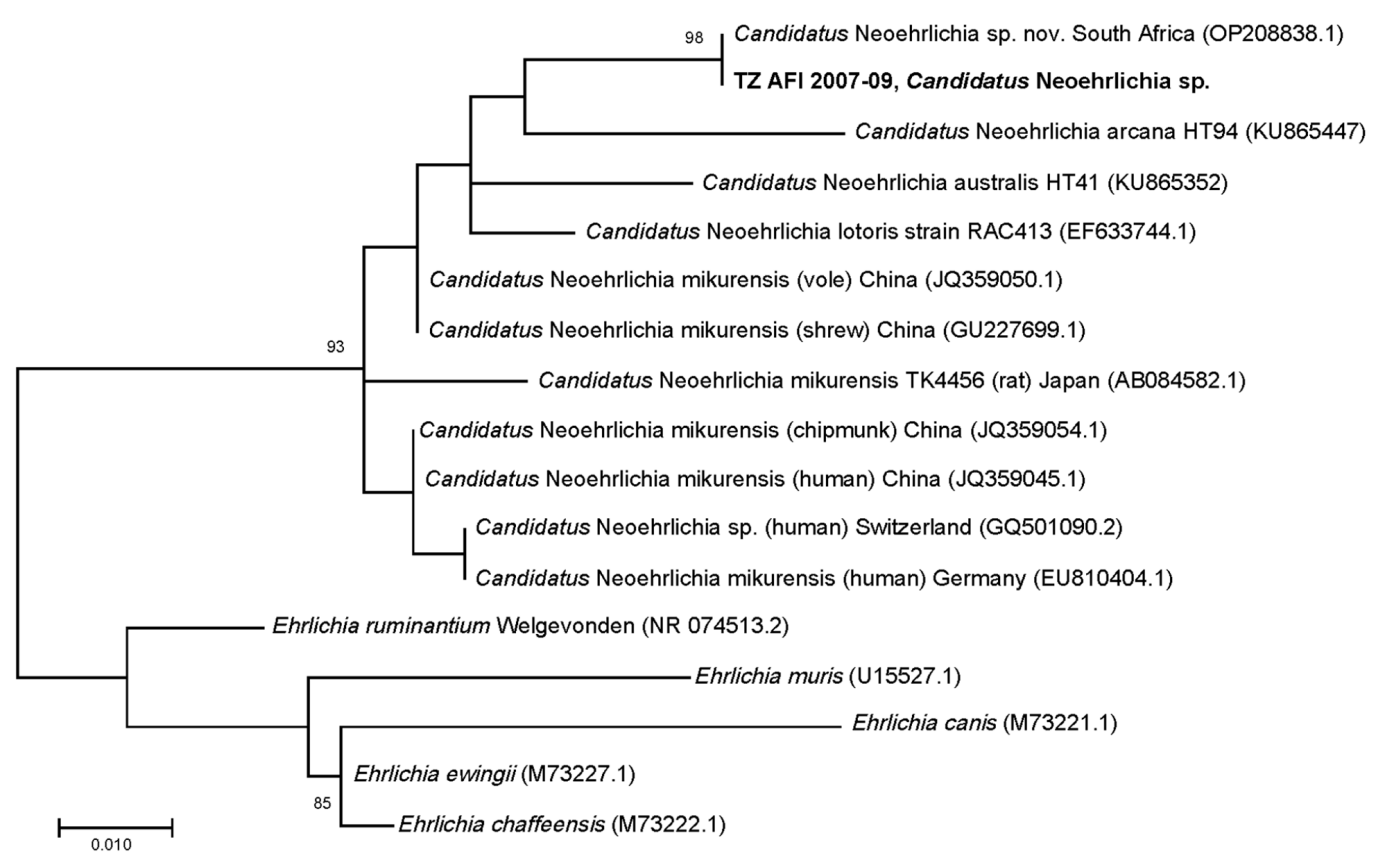
Phylogenetic tree for *Candidatus* Neoehrlichia spp. identified during metagenomic detection of bacterial zoonotic pathogens among febrile patients, Tanzania, 2007–2009. Bold text indicates the sequence from this study. Numbers in parentheses indicate GenBank accession numbers. A 1,467-bp 16S sequence amplified from a bone marrow aspirate from a patient from South Africa (GenBank accession no. OP208838) matched 100% over the 296-bp variable regions 1 and 2 target sequence amplified in this study (*18*). Scale bar indicates nucleotide substitutions per site.

We created a phylogenetic tree for the identified *Candidatus* Neoehrlichia spp. (Figure 1). A 1,467-bp 16S sequence amplified from a bone marrow aspirate from a patient from South Africa (GenBank accession no. OP208838) matched 100% over the 296-bp V1–V2 target sequence amplified in this study (*18*).

**Figure 2:**
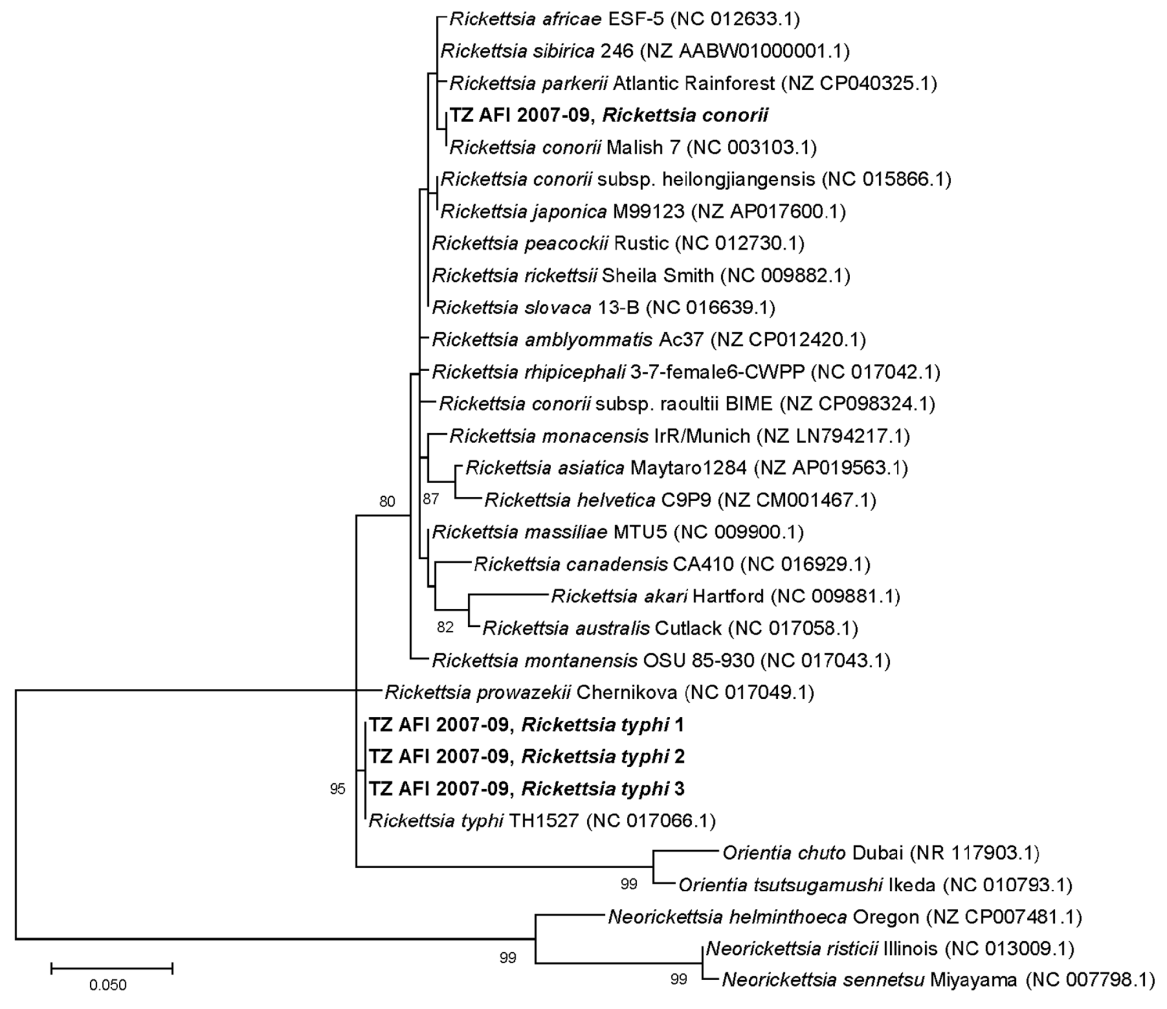
Phylogenetic tree of *Rickettsia* spp. sequences detected in metagenomic analysis of bacterial zoonotic pathogens among febrile patients, Tanzania, 2007–2009. The tree compares sequences from the 16S variable regions 1 and 2 (V1–V2) of the *Rickettsia* cohort from this study (bold text) to sequences from closely related *Rickettsia* species. Numbers in parentheses indicate GenBank accession numbers. The sequence from the study sample with *R. conorii* aligned 100% *R. conorii* strain Malish (accession no. NC003103.1) and was distinct from *R. africae* (accession no. NC012633.1). All 3 *R. typhi* strains from this study aligned 100% with *R. typhi* reference strain (accession no. NC017066.1) and were distinct from *R. prowazekii* (accession no. NC017049.1). The V1–V2 16S target is not sufficient to differentiate between *R. conorii* subsp. *heilogjiangensis* and *R. japonica*, or between *R. rickettsia*, *R. peacockii*, *R. philipii*, and *R. slovaca*. However, the V1–V2 16S target is sufficient to differentiate between *R. conorii conorii* and *R. africae* because 2 single-neucleotide differences would be expected between *R. conorii conorii* and *R. africae* and a TTT insertion in *R. africae*. Scale bar indicates nucleotide subsitutions per site.

We created a phylogenetic tree to compare the 16S V1–V2 of the *Rickettsia* sequences from this cohort to sequences from closely related *Rickettsia* species (Figure 2). The sequence from the study sample with *R. conorii* aligned 100% with *R. conorii* strain Malish (GenBank accession no. NC_003103.1) and was distinct from *R. africae* (accession no. NC_012633.1). The 3 *R. typhi* sequences all aligned 100% with *R. typhi* (accession no. NC_017066.1) and were distinct from *R. prowazekii* (accession no. NC_017049.1). The V1–V2 16S target is not sufficient to differentiate between *R. conorii* subspecies *heilogjiangensis* and *R. japonica*, nor among *R. rickettsia*, *R. peacockii*, *R. philipii*, and *R. slovaca*. However, that target is sufficient to differentiate between *R. conorii* and *R. africae* because 2 single-nucleotide differences are expected between those strains and *R. africae* has a TTT insertion.

**Figure 3:**
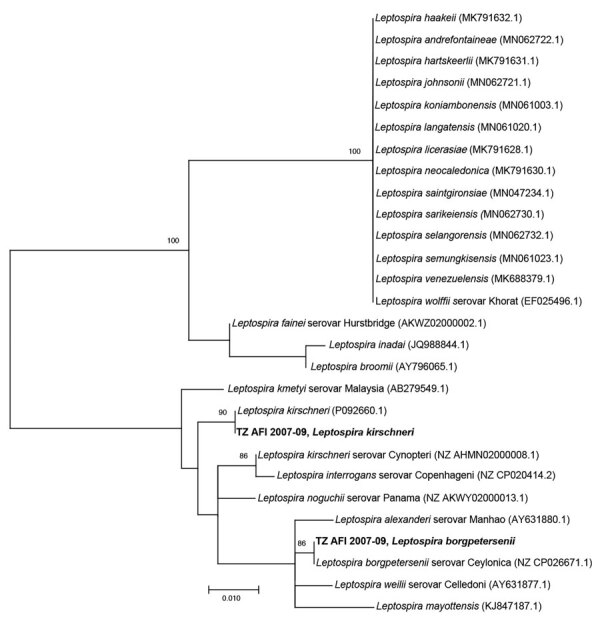
Phylogenetic tree of *Leptospira* sequences detected in metagenomic analysis of bacterial zoonotic pathogens among febrile patients, Tanzania, 2007–2009. The tree compares sequences from the 16S V1–V2 of the *L. kerchnerii* and *L.* *borgpetersenii* cohort from this study (bold text) to sequences from closely related *Leptospira* species. Numbers in parentheses indicate GenBank accession numbers. Scale bar indicates nucleotide subsitutions per site.

We detected *Leptospira* from 2 patients in the cohort. The phylogenetic tree comparing the 16S V1–V2 of the *Leptospira* sequences from this cohort to sequences from closely related species showed 1 *L. kirshneri* detection related to GenBank accession no. CP092660.1 and 1 *L. borgpetersenii* detection related to accession no. NZ_CP026671.1 (Figure 3).

We compiled serologic and PCR results of the 10 participants with bacterial zoonotic pathogen–positive samples (Table 1). Of those participants, 5 did not have serologic testing performed for the pathogen identified by 16S; 1 participant with detected *B. quintana* had serologic testing consistent with probable acute leptospirosis. The participant with *L. borgpetersenii* 16S detection had serologic testing consistent with acute leptospirosis and seroconverted to serogroup Mini, represented in the MAT panel by serovar Georgia. Real-time PCR *Leptospira* testing was not performed on samples from either of those participant. Two participants with *R. typhi* 16S detection had serologic evidence of exposure to typhus group *Rickettsia*, and 1 had serologic evidence of exposure to both spotted fever group *Rickettsia* and typhus group *Rickettsia* (Table 1).

## Discussion

Metagenomic sequencing on venous blood cell pellets from patients admitted with febrile illness in Tanzania generated several noteworthy findings, including detection of fleaborne or louseborne zoonotic pathogens, *B. quintana* and *R. typhi*; genetic confirmation of *R. conorii* in a febrile human from Tanzania, where spotted fever group *Rickettsia* is a common cause of febrile illness; and description of a potentially novel agent of neoehrlichiosis. 

Identification of *B. quintana* and *R. typhi*, both considered reemerging pathogens (*19*), are notable for northern Tanzania. In a study on *Rickettsia*, *Bartonella*, and *Yersinia* detected in fleas in 3 countries in Africa, *R. typhi* DNA was detected in 2 (2%) of 94 fleas collected in Tanzania, but *Bartonella* was not detected (*20*). However, multiple *Bartonella* species have been detected in fleas and small mammal samples in northern Tanzania (*21*). Despite those documented detections in fleas in Tanzania, the body louse is considered the primary vector for *B. quintana* (*22*). Consistent with the established clinical epidemiology of bartonellosis caused by *B. quintana*, 1 of the 2 *B. quintana* cases detected in our cohort occurred in an HIV-infected participant.

Detection of *R. conorii* in this cohort is a substantial public health finding because SFGR is a common cause of severe febrile illness in East Africa. Serologic testing is unable to distinguish among SFGR pathogens, including between *R. conorii* and *R. africae* (*23*). In a previous report, *R. africae* was detected from a patient with an eschar after travel to Tanzania (*24*). However, after comprehensive literature searches in multiple databases, extensive gray literature searching, and consultation with a reference librarian, we found no other examples of molecular detection of *R. conorii* in Tanzania. Genomic detection of *R. conorii*, the causative agent of Mediterranean spotted fever, is notable because *R. conorii* causes a more severe disease than *R. africae* and sometimes is fatal (*25*). Detection of *R. conorii* as a causative agent of SFGR in Tanzania is consistent with detections elsewhere in East Africa, including an *R. conorii* detection by PCR in a traveler returning to Japan from Kenya (*26*) and a report of fatal SFGR infection in Kenya in which the *Rickettsia* species was not identified (*27*). SFGR caused by *R. conorii* in East Africa is also supported by a domestic animal sampling study in which *R. conorii* subsp. *israelensis* was identified in domestic animals in Kenya and in ticks at slaughterhouses in Nairobi and Mombasa (*28*). Previous research in northern Tanzania has shown that SFGR is endemic (*4*,*8*). The identification of *R. conorii* as one of the agents of SFGR increases the severity profile of this disease in Tanzania and supports the need for advances in diagnostic testing for SFGR. In addition to the implications for SFGR disease burden due to severity and potential death, this finding also has implications for targeting disease prevention measures because *R. conorii* transmission would likely occur via *Rhipicephalus* ticks that infest canines, but *R. africae* transmission to humans would likely occur via *Amblyomma* ticks that infest cattle or other livestock (*29*,*30*).

The specimen with MiniKraken taxonomic predictions of Anaplasmataceae, *Ehrlichia ruminantium*, and *Ehrlichia* (Table 2) showed 100% sequence identity to a recently published novel *Candidatus* Neoehrlichia sp. sequence derived from a case of febrile illness in an immunocompetent child from South Africa who lived on a farm but had no reported tick bite (*18*). The 16S sequence from the patient from South Africa showed 100% identity to a 345-bp partial V3-V4 16S sequence (GenBank accession no. KT895260) derived from the blood culture of an patient from Austria with travel history to Tanzania who did not recall a tick bite during her trip but did have skin contact exposure to a prosimian (*31*). Those 2 *Candidatus* Neoehrlichia species infections and ours highlight the organism as a potential cause of a febrile illness in Africa. Further research is needed to determine illness severity, potential reservoirs, and geographic distribution.

Detection of pathogenic *Leptospira* spp. and *C. burnetii* is consistent with our prior work describing leptospirosis and acute Q fever as relatively common causes of febrile illness in northern Tanzania (*4*,*5*). Genetic characterization of both *Leptospira* spp. and *C. burnetii* associated with severe febrile illness in northern Tanzania are nonetheless notable findings and could aid in future studies investigating source attribution via genomic methods.

The participant with *L. borgpetersenii* seroconverted to serogroup Mini represented by serovar Georgia in the MAT panel. Few human leptospirosis infections in Tanzania have been genotypically characterized as *Leptospira* and our case had a combination of species and serogroup conversion (*32*). *L. kirschneri* was also detected from a febrile participant recruited in northern Tanzania during a 2012–2014 study. That participant seroconverted to serogroup Sejroe (*9*). Combined molecular and serologic data indicate multiple circulating *Leptospira* serovars in a small number of positive samples over 2 time periods. *Leptospira* diversity likely indicates complex transmission ecology in Tanzania with multiple serovars co-circulating in diverse hosts and circulating serovars changing over time (*33*).

One limitation of our study is that sensitivity of V1–V2 16S metagenomics was previously determined to be equivalent to that of RT-PCR in whole blood (*11*). However, the blood cell pellets used in this study represent a fraction of whole blood, a specific specimen type that has not been previously evaluated. In addition, although the overall percentage of samples positive by 16S metagenomics low (1.2%), that value was higher than the percentage (0.6%) of positive samples reported in a previous study that screened >10,000 specimens submitted for tickborne illness in the United States (*11*). Both studies relied on screening residual specimens; thus, the proportion of participants in whom bacterial sequencing detected a zoonotic pathogen does not give accurate indicators of disease prevalence.

In conclusion, by using targeted V1–V2 16S metagenomic testing among participants with febrile illness in Tanzania, we detected genera and some species of bacterial zoonoses that are of clinical and public health concern in northern Tanzania. The genetic confirmation of *R. conorii* detected here more broadly expands our understanding of the species responsible for SFGR in Tanzania and East Africa. 16S rRNA metagenomics also confirmed typhus group *Rickettsia*, *Leptospira*, and *C. burnetii* as causes of severe febrile illness in northern Tanzania. The assay also detected *Bartonella* and identified a potentially novel agent of neoehrlichiosis in sub-Saharan Africa. We demonstrated that metagenomic approaches can improve the etiologic and epidemiologic understanding of febrile illness. Although the overall low number of detections might preclude the 16S rRNA platform from being a standalone surveillance approach, our study highlights that this sequence-based approach provides genetic epidemiologic insights that can inform and optimize disease surveillance and prevention strategies.
